# *SF3B1* Mutations in Hematological Malignancies

**DOI:** 10.3390/cancers14194927

**Published:** 2022-10-08

**Authors:** Daniela Cilloni, Federico Itri, Valentina Bonuomo, Jessica Petiti

**Affiliations:** 1 Department of Clinical and Biological Sciences, University of Turin, 10043 Turin, Italy; 2 Division of Advanced Materials Metrology and Life Sciences, Istituto Nazionale di Ricerca Metrologica (INRiM), 10135 Turin, Italy

**Keywords:** *SF3B1*, hematological malignancies, splicing factor, spliceosome mutations, patient stratification

## Abstract

**Simple Summary:**

In recent years, spliceosome mutations have become of diagnostic and prognostic interest in several malignancies, as alternative splice mRNA isoforms are often associated with neoplasia. The role played by *SF3B1*, one of the splicing factors most frequently mutated in cancer, in different hematological neoplasia has been summarized here. A better knowledge of diagnostic and prognostic factors can allow a more precise stratification of hematological patients and a better prediction of the response to therapy.

**Abstract:**

Recently, mutations in the genes involved in the spliceosome have attracted considerable interest in different neoplasms. Among these, *SF3B1* mutations have acquired great interest, especially in myelodysplastic syndromes, as they identify a subgroup of patients who can benefit from personalized therapy. The *SF3B1* gene encodes the largest subunit of the splicing factor 3b protein complex and is critical for spliceosome assembly and mRNA splicing. The mutated *SF3B1* gene encodes for a protein with a different mRNA processing mechanism that results in the aberrant splicing of many mRNAs, which can be downregulated. Although there are many mRNAs affected by a splicing alteration, only a few of these have been directly related to the pathogenesis of several diseases. In this review, we took a snapshot of the current knowledge on the implications of *SF3B1* mutations in different hematological malignancies.

## 1. Introduction

In most coding genes, RNA splicing is essential for processing pre-mRNA. The main function of splicing is to remove non-coding introns and splice sequences (5′ss, BPS, and 3′ss) [1,2]. The spliceosome is the machinery that recognizes these latter sequences and catalyzes the splicing reaction. It is responsible for the excision of more than 99% of human introns. Most human genes encode several mRNA isoforms by the alternative splicing process. Because alternatively spliced mRNA isoforms are often associated with cancer, spliceosome mutations have recently sparked significant interest in different neoplasms, including hematological malignancies. Indeed, they are observed in myelodysplastic syndrome (MDS), myeloproliferative neoplasms (MPN), acute myeloid leukemia (AML), chronic lymphocytic leukemia (CLL), and chronic myelomonocytic leukemia (CMML).

Among the spliceosome mutations, those in the *SF3B1* gene are the most frequent and relevant in hematological diseases. The *SF3B1* gene is located on the long arm of chromosome 2 (2q33.1) and encodes for the biggest subunit (155 KDa) of the splicing factor 3b protein complex [3]. The interaction between the *SF3B1* complex, the 12S unit, and the splicing factor 3a complex gives rise to the U2 small nuclear ribonucleoproteins (snRNP), fundamental for spliceosome assembly and mRNA splicing [4]. The SF3B1 protein is located principally in the nucleus, where it forms nuclear speckles; it is necessary to anchor U2 snRNP to the pre-mRNA by sequence—independently binding the intron branch site [5].

**Figure 1:**
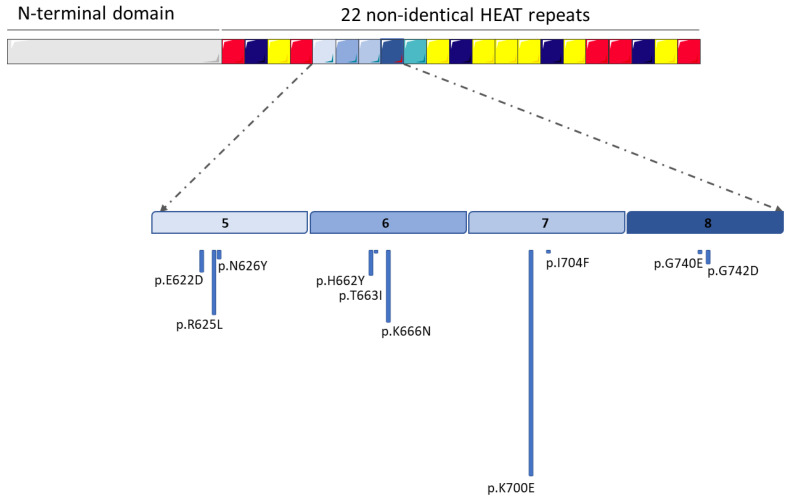
Schematic representation of the most frequent mutations of the *SF3B1* gene that characterize hematological neoplasms. The numbers 5, 6, 7 an 8 in the figure correspond to the non-identical HEAT repeats most frequently affected by mutations. (the figure was modified from Servier Medical Art, licensed under a Creative Common Attribution 3.0 Generic License, http://smart.servier.com/, accessed on 17 August 2022).

The major portion of the C-terminal domain of *SF3B1* is constituted by 22 non-identical HEAT repeats [6]. Almost all known mutations are found in domains from 4 to 12 (H4-H12), mostly on codon 700, which represents 50% of all of the known mutations; other mutations were found in codons 666, 662, 622, and 625 [7] (Figure 1).

**Figure 2:**
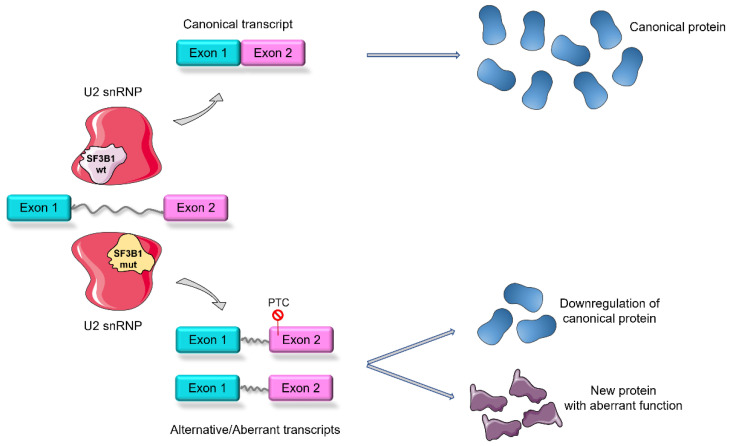
Schematic representation of *SF3B1* wild-type (wt)-mediated splicing, which generates canonical transcription and translation, and *SF3B1*-mutated (mut)-mediated splicing, which results in aberrant/alternative transcription. As a consequence, premature stop codons (PTC) can form, leading to the down-expression of canonical proteins or the translation of new proteins with aberrant functions (the figure was modified from Servier Medical Art, licensed under a Creative Common Attribution 3.0 Generic License, http://smart.servier.com/, accessed on 18 August 2022).

It is known that *SF3B1* mutations could play multiple roles in the pathogenesis of tumors by dysregulating several cellular functions and pathways, including heme biosynthesis, mitochondrial metabolism, and the NF-κB pathway [8]. Mutated *SF3B1* gives rise to a protein with a neomorphic activity linked to several mechanisms involved in RNA processing. About half of aberrantly spliced mRNAs are characterized by the presence of premature stop codons, resulting in nonsense-mediated RNA decay and downregulation of the protein expression. Although the wild-type allele is still able to be expressed, the amount of protein produced is unable to compensate for the mutant allele [9,10]. Another possibility is the translation of new proteins that lack the correct biological function and/or that acquire aberrant functions (Figure 2).

Mutated *SF3B1* involves the splicing alteration on a wide range of mRNAs. Nevertheless, only a small part of these has been identified as directly implicated in the disease pathogenesis.

Although the growing interest in spliceosome mutation effects, it is not yet clear what the functional role of the mutated SF3B1 protein is in the development of clonal hematopoiesis and risk for hematologic malignancies. To investigate the physiological function of *SF3B1*, several in vitro and in vivo models have been developed, including the generation of different *SF3B1* K700E-mutated mice models. Obeng and colleagues demonstrated that heterozygous expression of *SF3B1* K700E caused progressive macrocytic anemia [11]; conversely, Mupo and colleagues showed that mice develop progressive normocytic anemia without ring sideroblasts [12].

The growing interest in the mutational status of *SF3B1* has led to its incorporation into routine diagnostic practices. The most common methods used to investigate *SF3B1* genotype are Next Generation Sequencing (NGS) and Sanger Sequencing. The need for a massive characterization of patients has also stimulated the development of new alternative molecular assays, including the High-Resolution Melting Analysis [13] and the Peptide Nucleic Acids-PCR Clamping [14].

Here we summarized the different implications of *SF3B1* mutations in distinct hematological neoplasia.

## 2. *SF3B1* in Myelodysplastic Syndromes

From the WHO classification of 2016 [15], the presence of ring sideroblasts identifies a particular subgroup of MDS named myelodysplastic syndromes with ring sideroblasts (MDS-RS), characterized by iron deposits in the perinuclear mitochondria. MDS-RS are further divided into MDS-RS with single lineage dysplasia and MDS-RS with multilineage dysplasia.

*SF3B1* is altered in about 15–20% of all MDS patients, increasing to more than 80% in MDS with ring sideroblasts (RS) [16]. In recent years, the link between RS and *SF3B1* mutations in MDS has become so evident that the fourth WHO changed the criteria for diagnosing MDS-RS. Indeed, the definition of MDS-RS became the presence of at least 15% of sideroblasts in the bone marrow. Still, if an *SF3B1* mutation is identified, the diagnosis of MDS-RS might be made if RS comprises as few as 5% of nucleated erythroid cells. Recently, the 5th WHO changed the classification of MDS by defining genetic abnormalities, identifying MDS with low blasts and *SF3B1* mutation (MDS-SF3B1) as a distinct disease type that includes most MDS with ≥5% RS. The term MDS with low blasts and ring sideroblasts is now used to define cases with wild-type *SF3B1* and ≥15% RS [17].

The *SF3B1* mutations lead to the aberrant splicing of several mitochondrial iron metabolic genes, including *TMEM14C*, *PPOX*, and *ABCB7*. In particular, the mis-splicing of *ABCB7* has been proposed to drive ring sideroblasts formation in patients affected by myelodysplastic syndromes with *SF3B1* mutation [18]. The negative regulation of the *ABCB7* gene, a mitochondrial exporter of iron, seems to be an important event in the RS genesis. An integrative analysis of mutation and gene expression data in hematopoietic stem cells from MDS patients identified a correlation between *SF3B1* mutations and a reduction in *ABCB7* expression [19]. The mutated SF3B1 protein uses an alternative splice site at 3’ of the *ABCB7* pre-mRNA; consequently, the *ABCB7* alternative transcript contains a premature termination codon and is degraded, reducing its expression [20]. The results of the studies support a pathophysiological model in which the reduced ABCB7 expression leads to the mitochondrial iron accumulation observed in erythroblasts of patients with MDS-RS [21]. Furthermore, the overexpression of different heme biosynthesis enzymes, such as *SLC25A37*, which encodes a mitochondrial iron importer, and *GLRX5*, which encodes for a mitochondrial protein, was observed in CD34+ cells of MDS-RS patients with mutated *SF3B1* [22].

Interestingly, by making use of a model of iPS derived from an MDS patient with the *SF3B1* mutation, which recapitulates global mis-splicing observed in primary MDS-RS cells, Clough and colleagues gave rise to RS in vitro. They demonstrated that RS formation starts at the polychromatic stage and increases in the orthochromatic erythroblasts, thus reflecting a perinuclear distribution of mitochondria in late-stage erythroblasts [18]. Importantly, they demonstrated that reduced expression due to mis-splicing of mitochondrial transporters TMEM14C and ABCB7 causes RS formation, thus explaining the strong association between *SF3B1* mutations and RS in MDS.

Recently, Zhao and colleagues investigated the protein interaction between wild-type and K700E-mutated *SF3B1* and found that mutated *SF3B1* has a reduced interaction with two RNA helicases, DDX42 and DDX46. This study identified DDX42 as responsible for the aberrant splicing induced by *SF3B1* K700E [23].

Previous studies revealed that 35% of aberrantly spliced transcripts are more translated than their corresponding canonically spliced transcripts. This mostly occurs in genes with enriched metabolic functions. Interestingly, mutant *SF3B1* has been demonstrated to alter the expression of proteins affecting metabolic pathways. It is evident that mutant *SF3B1* reduces mitochondrial respiration and promotes glycolysis to compensate for defective mitochondrial metabolism, thus sensitizing cells to glycolysis inhibition. This could represent a promising therapeutic strategy [24].

From a clinical point of view, the presence of an *SF3B1* mutation appears to be an early event in MDS pathogenesis, manifests a distinct gene expression profile, and correlates with a favorable prognosis and a low risk of evolving into AML [25,26]. The treatment options for MDS-RS patients include supportive care [27], such as transfusions and/or erythropoietic stimulating agents (ESA); however MDS-RS are usually characterized by poor and short responses to ESA treatment [28].

Luspatercept (ACE-536) is a recombinant fusion protein consisting of a modified form of the extracellular domain of the human activin receptor type IIB (ActRIIB). The ActRIIB receptor and its ligands are members of the TGFβ superfamily. Luspatercept works by binding and inhibiting ligands of the TGFβ superfamily, among which the main one and the best defined is GDF11, and by interrupting their signaling, which is significantly increased in MDS. The luspatercept mechanism is independent of erythropoietin (EPO): while EPO stimulates the proliferation and differentiation of the first erythroid progenitors, luspatercept promotes the differentiation of precursors in the advanced stage. In a phase 2 study enrolling patients with lower-risk MDS, a high percentage of erythroid responses was observed. Sixty-three percent of luspatercept-treated patients obtained an erythroid improvement, and 38% obtained transfusion independence for at least 8 weeks [29]. Since the overall response rate was higher among patients with ring sideroblasts compared to the other subtypes of lower-risk MDS, a phase 3 trial, named MEDALIST, was designed to evaluate the efficacy of luspatercept in patients with lower-risk MDS-RS who were refractory or resistant to ESA. The trial demonstrated that this drug could be particularly effective in *SF3B1*-mutant MDS-RS [30]. Based on these data, luspatercept is now under investigation in another phase 3 trial that is currently exploring the efficacy of luspatercept head-to-head with erythropoietin in low-risk MDS patients for ESA naive patients [31].

## 3. *SF3B1* in Myeloproliferative Neoplasms

MPNs are a group of blood diseases with a dysregulated production of erythrocytes, leukocytes, and platelets. Based on the presence of *BCR-ABL1* fusion transcript, MPNs can be divided into Philadelphia positives and negatives (Ph-), which include polycythemia vera (PV), essential thrombocythemia (ET), and primary myelofibrosis (PMF). Most Ph- MPNs are characterized by alterations in three genes: *JAK2*, *MPL*, and *CALR* [15]. Nevertheless, a small percentage of them were mutated in *SF3B1* as an additional non-driver mutation: approximately 3–5% of PV and ET and 10% of PMF patients [32]. Heterozygous missense mutations were found in exons 14–16, and the K700E hotspot was the most frequent.

Unlike what happens in MDS, in MPNs the *SF3B1* mutations appear to increase the risk of fibrotic transformation [33,34]. It has also been suggested that the co-occurrence of *SF3B1* and *JAK2* or *MPL* mutations is associated with an increased risk of thrombotic events [35,36]. Despite this, data about the *SF3B1* mutations in Ph- MPNs are contradictory. Senin and colleagues described the association between *SF3B1* mutations and PV progression to MF [37]; conversely, Tefferi and colleagues did not confirm this result, but they correlated *SF3B1* mutations with decreased myelofibrosis-free survival in ET patients [38]. Successively, Boiocchi and colleagues suggested that *SF3B1* mutations occur earlier in PMF than in PV and ET. They also claimed that *SF3B1* mutations do not correlate with fibrotic evolution because the frequency in early/pre-fibrotic and advanced fibrotic stage PMF is similar [36]. Furthermore, current data on the clinicopathological features associated with *SF3B1* mutations in PMF are also ambiguous. In 2012, Lasho and colleagues found in their cohort that all PMF patients with an *SF3B1* mutation have high percentages of RS [32]; meanwhile, Boiocchi and colleagues observed that only a subgroup of the *SF3B1*-mutated patients showed RS [36].

Due to this contradictory evidence, at present *SF3B1* mutations do not have a clear prognostic significance in MPNs, and they still have no role in therapeutic decisions. However, this paradigm may also change soon, as a phase III study is underway to explore the use of luspatercept in MPNs, specifically in PMF and PPV-MF, and PET-MF (NCT04717414). In addition, a recent study conducted by Loscocco and colleagues investigated the impact of *SF3B1* mutations in secondary MF (SMF) with respect to PMF. They found that the *SF3B1* mutation did not impact overall survival (OS) in PMF, while it negatively affected OS in SMF cases [39].

## 4. *SF3B1* in Myelodysplastic/Myeloproliferative Neoplasms

Based on clinical and molecular characteristics, the WHO classification of 2016 identified MDS/MPN as an overlap syndrome with features of both MDS and MPN at the time of diagnosis [15]. MDS/MPN with ring sideroblasts and thrombocytosis (RS-T) is a distinct entity defined in the revised fourth edition of WHO [15]. The criteria for the diagnosis include thrombocytosis (≥450 × 109/L) associated with refractory anemia; dyserythropoiesis in the BM with ring sideroblasts accounting for 15% or more of erythroid precursors and megakaryocytes with features resembling those in PMF or ET; *SF3B1* mutation or, in the absence of *SF3B1* mutation, no history of recent cytotoxic or growth factor therapy that could explain the myelodysplastic/myeloproliferative features; no BCR-ABL1 fusion gene, no rearrangement of PDG-FRA, PDGFRB, or FGFR1; or PCM1-JAK2; no (3;3)(q21;q26), inv(3)(q21q26) or del(5q); no preceding history of MPN, MDS (except MDS-RS), or another type of MDS/MPN [15]. *SF3B1* mutations in MDS/MPN-RS-T occur in about 80% of patients but, unlike MDS, do not alter the number of RS required for the diagnosis [15]. Based on *SF3B1* status, the fifth WHO renamed MDS/MPN with RS as MDS/MPN with *SF3B1* mutation and thrombocytosis, while MDS/MPN with RS and thrombocytosis is used for cases with wild-type *SF3B1* and ≥15% RS [17]. Approximately 50% of patients harbored both the *JAK2* V617F and *SF3B1* mutations [40], while the co-occurrences of *SF3B1* and *CALR* or *MPL* mutations are about 1–3% [41]. In MDS/MPN overlap syndrome, *SF3B1* mutations seem to represent the founder mutations followed by secondary hits in genes involved in other signaling pathways, such as *JAK2* [42]. *SF3B1* mutations have been associated with an increased risk of thrombotic events and with an inferior thrombosis-free survival [35,43]. The mechanism by which *SF3B1* mutations might increase thrombotic risk in patients with MDS/MPN-RS-T has not yet been clarified.

Based on a post hoc analysis of the MEDALIST trial results, Luspatercept was also approved in 2019 for the management of anemia in patients with MDS/MPN-RS-T. Nevertheless, formal evidence about the safety and efficacy of this agent in MDS/MPN-RS-T has to be still assessed [31].

## 5. *SF3B1* in Chronic Myelomonocytic Leukemia

Chronic myelomonocytic leukemia (CMML) is a clonal stem-cell disorder characterized by absolute monocytosis with MDS/MPN features that mainly affects older adults.

*SF3B1* mutations are quite rare in CMML patients, detected in about 5–6% of the cases [7,44,45]. As described in MDS, the mutations are associated with the RS phenotype [45,46]. Wassie and colleagues have reported that *SF3B1* mutations correlate with an abnormal karyotype [47], while Patnaik and colleagues described a frequent association of *SF3B1* and *DNMT3A* gene mutations [43]. Furthermore, it was recently reported that *SF3B1*-mutated CMML has predominant dysplastic features, with a low frequency of *ASXL1* mutations and a higher frequency of *JAK2* mutations [48].

From a clinical point of view, CMML patients with *SF3B1* mutations show a lower white blood-cell count and low lymphocyte count respective to wild-type counterparts; however, this genotype does not seem to be associated with acute leukemic progression or to affect the overall survival [46,49].

## 6. *SF3B1* in Acute Myeloid Leukemia

AML is a malignant neoplasm characterized by defective hematopoietic stem-cell differentiation, apoptosis, and abnormal accumulation of immature myeloid blast cells. AML is the second most frequent hematologic malignancy in developed countries, representing about 30% of leukemia cases in adulthood [50]. *SF3B1* is mutated in 2–5% of all AML patients and in about 30% of AML-RS patients, most of whom are AML secondary to MDS. AML with these characteristics has been redefined as AML myelodysplasia-related (AML-MR) in the recent fifth WHO [17]. In AML-RS, *SF3B1* mutations are associated with higher age, normal karyotype, and a worse clinical outcome [51,52]. The correlation between *SF3B1* mutations and the RS phenotype is not as strong as in MDS. RNA sequencing analysis on precursor cells revealed that several heme metabolism-related genes are upregulated in AML-RS cells similarly to *SF3B1*-mutated MDS, demonstrating the involvement of common downstream effector pathways [53].

In AML, *SF3B1* mutations have been suggested as biomarkers of resistance to venetoclax [54], while they seem to confer sensitivity to decitabine-based therapy [55]. Furthermore, preliminary results of co-treatment with venetoclax and hypomethylating agents indicated comparable outcomes between the wild-type group and patients with mutations in spliceosome genes, including *SF3B1* [53]. Based on the paucity and inconclusiveness of the present data, it can only be concluded that further studies are needed to elucidate the clinical significance of *SF3B1* mutation in AML.

## 7. *SF3B1* in Chronic Lymphocytic Leukemia

Chronic Lymphocytic Leukemia (CLL) is the most common leukemia in the adult population; it is characterized by the increased proliferation of the B-lymphocytes that accumulate in the bone marrow, lymph nodes, and peripheral blood.

CLL is a clinically heterogeneous disease, as some patients progress rapidly toward more advanced studies, whereas others survive for a long period without the need for treatment. This heterogeneity of clinical course was somehow unexplained until studies on the CLL cell features disclosed that the CLL clones were heterogeneous and were characterized by different phenotypic and genotypic features in the different patients.

Mutations in the *SF3B1* gene were found in about 10–15% of CLL and correlate with deletions of 11q22 and *ATM* mutations, a poor prognosis, and resistance to fludarabine therapy [56,57,58]; nevertheless, there is no correlation between *SF3B1* and IGHV mutational status [59]. Rosenquist and colleagues reported a comparable poor outcome in patients with *SF3B1* and *TP53* mutations [60], and Wang and colleagues suggested that *SF3B1* status could represent an independent risk factor for CLL [56]. Furthermore, the *SF3B1* mutation rate was higher in patients previously treated with chemotherapy than in untreated subjects, suggesting that, unlike other nonsynonymous mutations in CLL, the *SF3B1* mutations could be induced or selected by chemotherapy [61]. Interestingly, mutations of *SF3B1* are detected more frequently than those of *BTK* in patients who have developed resistance to ibrutinib, a specific *BTK* inhibitor [62]. Furthermore, Pozzo and colleagues recently demonstrated that *SF3B1* mutations correlate with increased activation of the NOTCH1 signaling due to the over-expression of an alternatively spliced form of DVL2. It also described a downregulation of surface CD20 antigen in *SF3B1*-mutated CLL, thus providing a rationale for using novel therapeutic schemes without the addition of anti-CD20 molecules in these patients [63].

Currently, the impact of *SF3B1* mutations at the cellular level remains unclear in CLL. Quesada and colleagues reported relatively few transcripts with altered splicing in CLL patients with mutated *SF3B1* [57]. One of the identified variations associated with *SF3B1* mutations is the expression of a truncated *FOXP1* mRNA, that is implicated in diffuse large B-cell lymphoma pathogenesis [64]. Furthermore, Raa and colleagues demonstrated that CLL patients with *SF3B1* mutations have a decreased expression of TP53 target genes after irradiation, highlighting the possible role of *SF3B1* mutations in regulating responses to DNA damage [65].

For therapeutic purposes, different in vitro studies have evaluated the effects of splicing modulators on *SF3B1*-mutated CLL cells. In particular, FD-895 and pladienolide B have been observed to rapidly induce a process of intron retention and strong apoptosis. Furthermore, their pro-apoptotic activity was not observed in normal lymphocytes [66]. This evidence paves the way for the development of new target therapies in *SF3B1*-mutated CLL.

## 8. Conclusions

Given the recent update in the WHO guidelines [17], the assessment of the mutational status of *SF3B1* is becoming increasingly important. In MDS and MDS/MPN, detecting these mutations is relevant for diagnostic and therapeutic purposes. The information about the mutational status of *SF3B1* has not only allowed the redefinition of the diagnostic criteria in MDS but also the improvement of the risk stratification, identifying new sub-categories: MDS-SF3B1 and MDS/MPN with *SF3B1* mutation and thrombocytosis. Especially in MDS, improving knowledge about the genetic profile is fundamental, given the recent development of a new International Prognostic Scoring System-Molecular (IPSS-M), which include somatic gene mutation data to hematologic parameters and cytogenetic abnormalities. Precisely in this context, *SF3B1* mutations were associated with favorable outcomes, modulated by a pattern of co-mutations [67]. In the setting of AML and CLL, although the evidence is not so strong, the possible therapeutical implications of *SF3B1* mutations are intriguing for the future development of new target drugs. Furthermore, the *SF3B1* genotype, together with the mutational status of the other seven genes and the cytogenetic and molecular characteristics, can contribute to diagnosing AML-MR. In MPN, this topic is still under investigation because the available data are too contradictory and inconclusive.

Many steps have been taken in understanding the biological role of these mutations, and the time when this mutation will be targetable is coming closer and closer. The great achievement of the last few years will soon allow a more precise stratification of the hematological patient and a prediction of response to therapy. Therefore, the road to better characterize the large family of hematological neoplasms continues, which is increasingly emerging as a larger and more heterogeneous group of diseases than was thought only a few years ago.

Furthermore, the possibility of obtaining the mutational status of *SF3B1*, with methods that are increasingly sensitive, specific, and inexpensive in terms of knowledge, time, and money, will allow its dissemination outside the reference centers.
